# Environmental bisphenol A exposure triggers trained immunity-related pathways in monocytes

**DOI:** 10.3389/fimmu.2023.1270391

**Published:** 2023-11-23

**Authors:** Marcello Dallio, Lorenzo Ventriglia, Mario Romeo, Flavia Scognamiglio, Nadia Diano, Martina Moggio, Marina Cipullo, Annachiara Coppola, Athanasios Ziogas, Mihai G. Netea, Alessandro Federico

**Affiliations:** ^1^Hepatogastroenterology Division, Department of Precision Medicine, University of Campania “Luigi Vanvitelli”, Naples, Italy; ^2^Department of Experimental Medicine, University of Campania “Luigi Vanvitelli”, Naples, Italy; ^3^Department of Internal Medicine and Radboud Center for Infectious Diseases (RCI), Radboud University Nijmegen Medical Centre, Nijmegen, Netherlands; ^4^Department of Immunology and Metabolism, Life and Medical Sciences Institute (LIMES), University of Bonn, Bonn, Germany

**Keywords:** trained immunity, bisphenol A, endocrine-disrupting compounds, innate response, inflammation

## Abstract

**Introduction:**

Trained Immunity represents a novel revolutionary concept of the immunological response involving innate immune cells. Bisphenol A is a well-known endocrine disrupter, widely disseminated worldwide and accumulated in the human body. Due to the increased interest regarding the effects of plastic-derived compounds on the immune system, our purpose was to explore whether BPA was able to induce trained immunity in human primary monocytes *in vitro* using low environmental concentrations.

**Materials and methods:**

We extracted BPA from the serum of 10 healthy individuals through a liquid-liquid extraction followed by a solid phase extraction and measured the concentration using an HPLC system coupled to a triple quadrupole mass spectrometer. In parallel, monocytes were isolated from whole blood and acutely stimulated or trained with BPA at three different concentrations (1 nM, 10 nM, 20 nM). Pro- and anti-inflammatory cytokines (IL-1β, TNF-α, IL-6, and IL-10) production were assessed after 24 hours of acute stimulation and after Lipopolysaccharide (LPS) rechallenge. A comprehensive overview of the metabolic changes after BPA acute stimulation and trained immunity induction was assessed through extracellular lactate measurements, Seahorse XFb metabolic flux analysis and ROS production.

**Results:**

Monocytes primed with BPA showed increased pro- and anti-inflammatory cytokine responses upon restimulation, sustained by the modulation of the immunometabolic circuits. Moreover, we proved the non-toxic effect of BPA at each experimental concentration by performing an MTT assay. Additionally, correlation analysis were performed between pro- and anti-inflammatory cytokines production after LPS acute stimulation or BPA-mediated trained immunity and BPA serum concentrations showing a significant association between TNF-α and BPA circulating levels.

**Discussion:**

Overall, this study pointed out for the first time the immunological effects of an environmental chemical and plastic-derived compound in the induction of trained immunity in a healthy cohort.

## 1 Introduction

In the last decades, an increasing number of studies assessing adaptive mechanisms within the innate immune system have described long-term functional changes in innate immune cells after an insult, a de-facto innate immune memory that was termed *trained immunity* (TI) (1–3). After exposure to certain infections or vaccines, innate immune cells appear able to react more strongly to a second stimulation in an antigen-agnostic manner: this is functionally equivalent to a memory response, not described previously by the scientific community (2–4). At a molecular level, TI is induced by the activation of different signaling pathways that lead to epigenetic remodeling and consequently, the rewiring of intracellular metabolic pathways, determining the acquisition of a long-lasting and self-regulating phenotype associated with an increase of pro-inflammatory cytokine production (5). The immunological phenotype of TI has been proven to last up to 1 year, although heterologous protection against infections induced by live vaccines has been documented for longer periods (6). While induction of TI constitutes a natural defense response in infections, an inappropriate TI induction may contribute to the onset and worsening of several chronic inflammatory diseases (5).

Endocrine-disrupting compounds (EDCs) are a heterogeneous class of molecules mimicking, blocking, or interfering with hormone signaling, implicated in the pathogenesis of various human diseases (7). Bisphenol A (BPA) is a synthetic organic EDC with a molecular weight of 228 Da and chemical formula (CH_3_)_2_C(C_6_H_4_OH)_2_ included in the group of diphenylmethane and bisphenol derivatives (8). It is mainly used for the production of polycarbonate plastic, whose wide diffusion is responsible for its ubiquitous human exposure (9). Alarmingly, BPA chronic ingestion and dermal absorption have been associated with different immunological and metabolic human disorders, representing thus a global health concern (8–10). From a pathogenetic point of view, BPA exposure may determine pleiotropic effects. First, it promotes systemic oxidative stress by increasing the production of reactive oxygen species (ROS), mainly through the blockade of the cytochrome P450 enzyme complex, as well as by inhibiting antioxidant genes expression such as superoxide dismutase (SOD), catalase (CAT), and reduced glutathione (GSH), playing a pivotal role in the maintenance of redox and immunological balance (11, 12). Second, BPA can directly influence gene transcription through epigenetic regulation by DNA methylation, histone modifications, and microRNA (miRNA) profile alterations (10). Finally, BPA can alter metabolic homeostasis, influencing glucose and lipid metabolism, through the activation of various inflammatory pathways (9, 13).

Considering the strong immunological impact and the widespread dissemination of environmental BPA, in this study we aimed to explore the role of BPA as a novel triggering stimulus of TI in primary cultured monocytes.

## 2 Materials and methods

### 2.1 Individuals’ enrollment and sample preparations

Ten healthy individuals of normal weight (6 males and 4 females; ranging-age: 24-52 years old) were recruited in compliance with the ethical guidelines of the Helsinki Declaration (1975) and after the approval of the ethical committee of the University of Campania “Luigi Vanvitelli” in Naples (protocol N17234/2022). All the subjects were recruited in the geographic area of Naples (Campania Region).

From each participant, written informed consent was obtained, and subsequently, 25 mL of peripheral blood sample was collected. Ten mL of blood were harvested in sodium-citrate BPA-free tubes (BD biosciences, USA) to obtain serum for BPA extraction and quantification, while 15 mL were placed in ethylene-diamine-tetra-acetic acid (EDTA) tubes (BD biosciences, USA) for peripheral blood mononuclear cells (PBMCs) and monocytes isolation.

**Figure 1:**
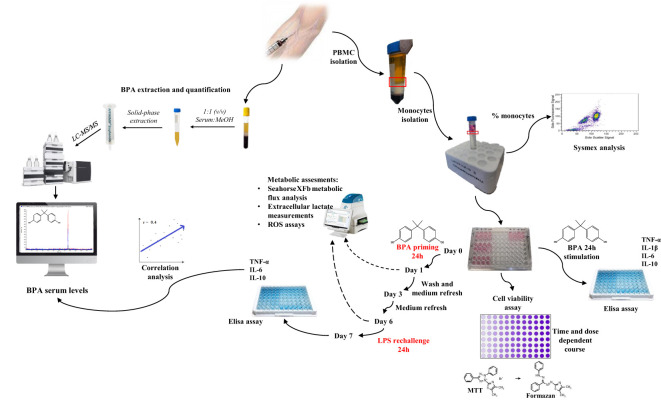
Summarized experimental setup.

The entire amount of blood was immediately used after the collection for the specific investigations. The entire experimental setup is summarized in **Figure 1**.

### 2.2 Bisphenol A extraction and LC-MS/MS analysis

The blood collected in sodium-citrate BPA-free tubes was immediately centrifuged for 15 minutes at 2000 rpm. After centrifugation, the serum was separated from the corpuscular part and placed in 15 mL glass tubes. Detailed BPA analysis methods have been published by Nicolucci et al., (14) including the quality control system used to monitor method performance and to prevent analysis contamination. Briefly, serum samples were undergone to liquid-liquid extraction of BPA with methanol (1:1, v/v) and to solid-phase extraction cartridge (AFFINIMIP Bisphenols, Polyntell SA, Paris, France) for the clean-up and concentration. The analysis of sample extracts was carried out by a DionexUltiMate 3000 High-Performance Liquid Chromatography (HPLC) system coupled to a triple quadrupole mass spectrometer (API 2000; AB Sciex, Germany).

A Kinetex F5 (100 x 4.6 mm, 2.6 µm) stainless-steel column (Phenomenex, Italy) was used for reversed-phase separation.

Chromatography was run at room temperature by linear gradient elution in water and methanol. The analytes were quantified in a multiple-reaction monitoring mode, according to Nicolucci et al., 2017.

All samples were analyzed in triplicate with their relative standard deviations (RSDs), less than 13% (14).

### 2.3 PBMCs and monocytes isolation

The isolation of PBMCs was performed starting with the dilution of whole blood samples (15 mL) of each subject in Phosphate-buffered saline (PBS) and density centrifugation over Lymphoprep™ (STEM CELL™ TECHNOLOGIES, Germany).

Cells were washed three times in cold PBS and afterward resuspended in a warm RPMI 1640 (with L-glutamine) culture medium (Life Technologies, Italy). Percoll isolation of monocytes was performed according to Domìnguez-Andrès et al., protocol (15). Briefly, previously isolated PBMCs were layered on top of a hyperosmotic Percoll solution (48.5% Percoll [Cytiva, Sweden], 41.5% sterile H_2_O, and 10% of 0.22 µM filter-sterilized 1.6 M NaCl) and centrifuged for 15 minutes at 580 x g, 20°C, acceleration 1, no break. The interphase layer was isolated, and the cells were washed with cold PBS. Cells were resuspended in warm RPMI 1640 (with L-glutamine) culture medium supplemented with 10% Foetal Bovine Serum (FBS, Life Technologies, Italy), 50 µg/mL gentamycin (Sigma-Aldrich, USA) and 1 mM sodium pyruvate (Sigma-Aldrich, USA) [RPMI+] and counted.

To guarantee adequate purity, the Percoll-obtained monocytes were plated and incubated for 1 h at 37°C to let them appropriately adhere to the well of a 96-polystyrene flat-bottom plate (Thermofisher, Italy). The monocytes were then washed with warm PBS with calcium and magnesium (Life technologies, Italy) to obtain maximal purity avoiding T-cell contamination and kept in culture in RPMI+.

The efficiency of monocyte isolation according to this protocol was established at 95% as described by Domìnguez-Andrès et al. (15).

### 2.4 Sysmex analyses

To further confirm the identity of isolated monocytes, 25 µL of the enriched suspensions were analyzed with an XN Sysmex hematology analyzer (Sysmex, Kobe) to count and obtain monocyte percentages. The density plots are based on the side scatter signal and side fluorescence signal, while histograms are on the side scatter signal and event count.

### 2.5 BPA acute stimulation

Monocytes of each individual were seeded in duplicate (100,000 cells/well) in a flat-bottom 96-well plate. Cells were washed with warm PBS with calcium and magnesium after 1h of incubation at 37°C, 5% CO_2,_ and incubated with three different concentrations of BPA- 1 nM, 10 nM, 20 nM – (Sigma Aldrich, Italy), Lipopolysaccharide (LPS) 10 ng/mL (O111:B4, Sigma Aldrich, Italy) as a positive control and RPMI+ as negative control (in 200 µL/well). After 24 hours, supernatants were collected and centrifuged for 5 minutes at 500 x g avoiding the presence of residual debris and stored at -20°C until cytokine measurements.

### 2.6 Trained immunity protocol induction using BPA as a training stimulus

Monocytes of each individual were seeded in duplicate (100,000 cells/well) in a flat-bottom 96-well plate. Cells were washed with warm PBS with calcium and magnesium after 1h of incubation at 37°C, 5% CO_2,_ and incubated with three different concentrations of BPA- 1 nM, 10 nM, 20 nM – (Sigma Aldrich, Italy) and RPMI+ as negative control (in 200 µL/well). After 24 hours, the stimulation was removed by washing each well with warm PBS with calcium and magnesium, and fresh culture medium RPMI+ was added to the monocytes and incubated for 48 h at 37°C, 5% CO_2_. At day 3, the medium has been refreshed and monocytes were incubated for another 48 hours to let them rest for 6 days after the first stimulation. Cells were restimulated at day 6 with 10 ng/mL LPS (O111:B4, Sigma Aldrich, Italy) for 24 hours or left unstimulated. On the last day of protocol (day 7), supernatants were collected and centrifuged for 5 minutes at 500 x g avoiding the presence of residual debris and stored at -20°C until cytokine measurements.

### 2.7 Cytokine measurements

Cytokine measurement in supernatants was assessed using commercial enzyme-linked immunosorbent assay kits for Tumor necrosis factor alpha (TNF-α; BD biosciences), Interleukin 1β (IL-1β, Life technologies), Interleukin 6 (IL-6, BD biosciences), and Interleukin-10 (IL-10, BD biosciences) following the instruction of manufacturers.

### 2.8 Extracellular lactate measurements

Lactate concentrations in cell culture supernatants collected after 24h and 6 days were quantified using Amplex® Red reagent (10- acetyl-3,7-dihydroxyphenoxazine, 0.2 mM, Thermo Fisher Scientific). First, lactate oxidase (2 U/mL, derived from Aerococcus viridans, Sigma Aldrich) was used to break down lactate, yielding hydrogen peroxide (H2O2). In the presence of horseradish peroxidase (0.2 U/mL HRP, Thermo Fisher Scientific), hydrogen peroxide reacts with Amplex® Red to generate the fluorescent product resorufin. Because the oxidase- and peroxidase-mediated reactions are coupled, the amount of fluorescence directly correlates to the amount of lactate. Cell-free medium samples, incubated for the same amount of time, were included to allow for background correction. Fluorescence was measured (Ex: 570 nM, Em: 585 nM) and concentrations were derived from a standard curve of sodium-L-lactate (Sigma Aldrich).

### 2.9 Seahorse XFb metabolic flux analysis

Percoll-isolated monocytes (1 × 10^6^) were seeded into Seahorse XF cell culture plate and acutely stimulated for 24h with BPA for the first time point analysis. 10^7^ monocytes were cultured in 10 cm tissue culture plates (VWR) and BPA training protocol was applied as described before. At day 6, macrophages were detached with PBS+EDTA 2 mM and counted. 105 cells were seeded into Seahorse XF cell culture plate and incubated for 1 h at 37 °C, 5% CO2. After adhering for 1 h, the medium was changed to Seahorse XF assay medium pH 7.4 (Agilent) supplemented with 1 mM L-glutamine for Seahorse XF Glycolysis Stress Test or 2 mM L-glutamine, 11 mM D-glucose, and 1 mM pyruvate for Seahorse XF Cell Mito Stress Test. Cells were incubated in a non-CO2-corrected incubator at 37°C for 1 h. Oxygen consumption rate (OCR) was measured using Seahorse XF Cell Mito Stress Test, with final concentrations of 1 μM oligomycin (Sigma-Aldrich), 10 μM FCCP (1 carbonyl cyanide‐4‐(trifluoromethoxy) phenylhydrazone, Sigma-Aldrich), and 0.5 μM Antimycine A/Rotenone (Sigma-Aldrich). Extracellular acidification rate (ECAR) was measured using Seahorse XF Glycolysis Stress Test, with final concentrations of 11 mM D-glucose (Sigma-Aldrich), 1 μM oligomycin (Sigma-Aldrich), and 22 mM 2-DG (2-Deoxy-D-glucose, Sigma-Aldrich). All the measurements were carried out in quadruplicate or quintuplicate using an XFp Analyzer (Seahorse Bioscience).

### 2.10 ROS assay

Superoxide anion levels were detected using luminol-enhanced chemiluminescence and determined in a luminometer (Biotek Synergy HT). 10^5^ monocytes were incubated with BPA 1 nM or RPMI alone in a tissue culture white plate (Corning) for 24 h. ROS levels were measured after 24h or 6 days following opsonized zymosan (10 mg/mL) restimulation. Luminol (5-Amino-2,3-dihydro-1,4-phthalazinedione, Sigma Aldrich) was added to each well in order to start the chemiluminescence reaction. Each measurement was carried out in quadruplicates. Chemiluminescence was determined every 145 s at 37°C for 1h. Luminescence was expressed as relative light units (RLU) per second and as area under the curve (AUC).

### 2.11 Cell viability assay

Cell viability was determined on human primary monocytes using thiazolyl blue tetrazolium bromide [3-(4,5-dimethylthiazol-2-yl)-2,5-di-phenyltetrazolium bromide] (MTT; Sigma-Aldrich, Schnellendorf, Germany) assay, following the manufacturer’s instructions. A total of 1 × 10^4^ cells/well were plated in a 96-well plate and then treated with BPA; experiments were performed in triplicates and repeated for three different healthy individuals (S01, S02, S03). Absorbance was read at a wavelength of 570 nm with a TECAN INFINITE M PLEX reader (Tecan, Austria).

### 2.12 Statistical analysis and data availability

Continuous data were described as mean and standard deviations, while categorical variables were summarized as n (%). The Kolmogorov-Smirnov test for normality was performed to evaluate if parametric or non-parametric analysis should be applied. Wilcoxon signed ranks test, t-test for dependent groups, the Kruskal-Wallis test or ANOVA test with posthoc Tukey analysis, in the case of non-normal or normal distribution respectively, were performed to compare the continuous variables. Statistical significance was defined as p < 0.05 in a two-tailed test with a 95% confidence interval.

Pearson correlations were used to test the strength of the association between cytokines production of LPS acute stimulated monocytes or BPA-trained macrophages and BPA serum levels.

The analysis was performed using the R statistical software (version 4.3.0) with the *cor. test* function of the *stats* package. A *p-value* < 0.05 was considered to indicate statistical significance.

Cytokine production and viability data were analyzed and plotted with GraphPad Prism software version 8.4.3. Pearson correlations were analyzed and plotted with R.

All data and materials used in the analysis are available upon reasonable request for collaborative studies regulated by materials/data transfer agreements (MTA/DTAs) to the corresponding author.

## 3 Results

### 3.1 LC-MS/MS and BPA acute stimulation

**Figure 2:**
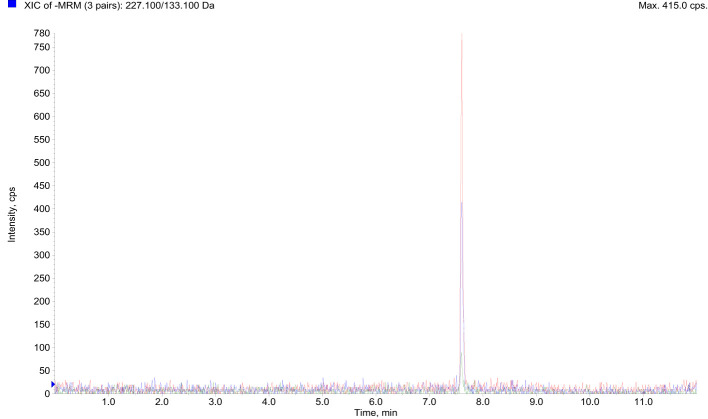
LC-MS/MS data showed BPA accumulation in the serum of 10 healthy donors. *BPA, Bisphenol A; LC-MS/MS, Liquid chromatography-mass spectrometry*.

The LC-MS/MS analysis on the serum of healthy donors demonstrated a low mean (16) BPA concentration of 0.148 ng/mL ± 0.084 (0,65 nM ± 0,37) for the analyzed population (**Figure 2** and **Table 1**).

**Table 1:** LC-MS/MS data showed BPA accumulation in the serum of 10 healthy donors.

Donors	Serum BPA concentration (ng/mL)	Serum BPA concentration (nM)
DONOR_A	0.1	0.44
DONOR_B	0.157	0.68
DONOR_C	0.172	0.75
DONOR_D	0.148	0.65
DONOR_E	0.204	0.89
DONOR_F	0	0
DONOR_G	0.238	1.04
DONOR_H	0.229	1.00
DONOR_I	0.019	0.083
DONOR_L	0.213	0.93

BPA, Bisphenol A; LC-MS/MS, Liquid chromatography-mass spectrometry.

**Figure 3:**
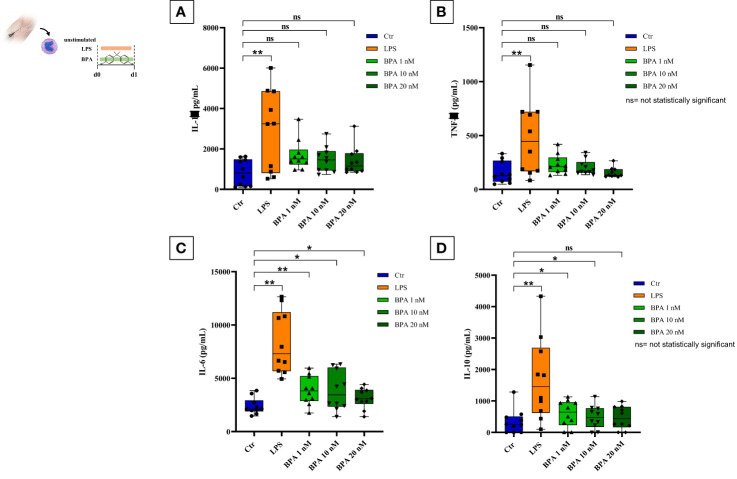
BPA acute stimulation. Pro-inflammatory **(A–C)** and anti-inflammatory **(D)** cytokines production after BPA 1 nM, 10 nM, and 20 nM acute stimulation (Wilcoxon signed ranks test *p<0.05; **p<0.005). *BPA, Bisphenol A*. ns, not statistically significant.

After the isolation of the enriched monocyte suspensions, their identity was further confirmed (**Supplementary Figure 1**) with a mean purity percentage of 70.35%. However, the purity increases after the 1-hour washing step as previously demonstrated by Bekkering et al., (17). Acute stimulation with LPS and BPA showed an increase in pro- and anti-inflammatory cytokines production compared to the baseline levels (**Figure 3**). Particularly, LPS stimulation (positive control) strongly induced the production of pro-inflammatory cytokines such as IL-1β (2931.70 pg/mL ± 2020.71) (**Figure 3A**), TNF-α (477.46 pg/mL ± 345.94) (**Figure 3B**), and IL-6 (8380.21 pg/mL ± 2950.11) (**Figure 3C**), along with the anti-inflammatory cytokine IL-10 (1691.38 pg/mL ± 1313.31) (**Figure 3D**) showing statistically significant differences with baseline cytokine levels (p<0.005).

BPA acute stimulation at the three different concentrations slightly induced the production of IL-1β (BPA 1 nM: 1696.29 pg/mL ± 754.54; BPA 10 nM: 1479.04 pg/mL ± 635.55, BPA 20 nM: 1402.97 pg/mL ± 707.20) (**Figure 3A**) and TNF-α (BPA 1 nM: 231.59 pg/mL ± 90.40; BPA 10 nM: 204.07 pg/mL ± 69.56, BPA 20 nM: 156.04 pg/mL ± 46.73) (**Figure 3B**) resulting in not statistically significant differences with baseline cytokine levels.

Conversely, IL-6 production appeared highly expressed in all BPA concentrations (BPA 1 nM: 3862.15 pg/mL ± 1341.71; BPA 10 nM: 3704.13 pg/mL ± 2087.22, BPA 20 nM: 3118.94 pg/mL ± 945.17) (**Figure 3C**) with a statistically significant difference compared to baseline levels. BPA acute stimulation induced a raise of anti-inflammatory IL-10 as well, but only BPA 1 nM (600.95 pg/mL ± 419.35) and BPA 10 nM (486.87 pg/mL ± 365.51) reached the statistically significant difference compared to the baseline levels, while the difference with BPA 20 nM (469.69 pg/mL ± 361.60) resulted not statistically significant (**Figure 3D**).

### 3.2 Induction of pro-inflammatory cytokine production consequent to BPA-trained stimulation

**Figure 4:**
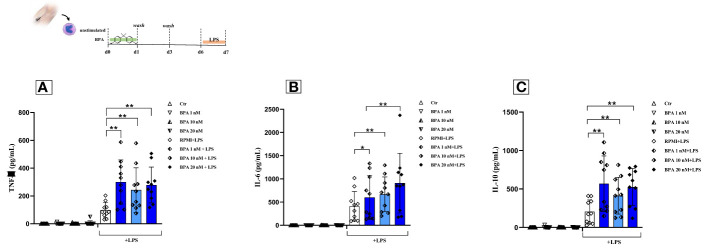
BPA-induced trained immunity and compensation mechanisms in healthy individuals. Pro-inflammatory and anti-inflammatory cytokines [Tumour necrosis factor alpha **(A)**; Interleukin-6 **(B)** and *Interleukin-10***(C)**] production after BPA 1 nM, 10 nM, and 20 nM training (Wilcoxon signed ranks test *p<0.05; **p<0.005). *BPA, Bisphenol A; TNF-a, Tumour necrosis factor alpha; IL-6, Interleukin-6; IL-10, Interleukin-10; Ctr, controls; LPS, Lipopolysaccharide*.

Monocytes were exposed only for the first 24 hours to three different concentrations of BPA (1 nM, 10 nM, 20 nM) and subsequently treated with LPS, as a second unrelated stimulus on day 6. This treatment induced a trained immune phenotype characterized by increased production of pro-inflammatory cytokines (TNF-α, IL-6) as shown in **Figure 4** (4A and 4B). Our data highlighted the establishment of a trained phenotype response to each BPA concentration chosen in combination with the LPS heterologous stimulus. Notably, our data showed that the pre-treatment with BPA 1 nM induced the highest output of TNF-α (BPA 1nM: 300.06 pg/mL ± 158.79) followed by BPA 10 nM and 20 nM (BPA 10 nM: 242.71 pg/mL ± 159.58; BPA 20 nM: 278.62 pg/mL ± 128.85), after secondary stimulation with LPS. All the concentrations determined statistically significant differences (p<0.005) between BPA-trained cells and LPS-stimulated cells in TNF-α production (**Figure 4A**).

Similar results were found analyzing the production of IL-6. The most triggering BPA concentration was 20 nM with an output of 918.65 pg/mL ± 648.24 compared to an amount of 626.42 pg/mL ± 494.45 and 726.25 pg/mL ± 422.89 in BPA 1nM and 10 nM respectively. All the IL-6 concentrations showed statistically significant differences (BPA 1 nM p<0.05; BPA 10 nM and 20 nM p<0.005) between BPA-trained cells and non-trained cells after secondary stimulation with LPS (**Figure 4B**). The same trend was still detectable at half of the median serum concentration (**Supplementary Figure 2**). The significant rise of pro-inflammatory cytokines argues that BPA could be considered a TI-inducing agent.

### 3.3 BPA-induced trained immunity determines an increased production of anti-inflammatory cytokine IL-10

A build-up in the production of the anti-inflammatory cytokine IL-10, upon the second stimulation with LPS at day 6 was also observed (**Figure 4C**). The powerful anti-inflammatory response was induced after secondary stimulation with LPS after pre-exposure to 1 nM of BPA exposure (568.05 pg/mL ± 361.93), although a trained phenotype was displayed also with the other BPA concentrations (BPA 10 nM: 412.89 pg/mL ± 239.74; BPA 20 nM: 525.94 pg/mL ± 239.72; all p<0.005).

### 3.4 BPA triggers changes in the immune metabolism

**Figure 5:**
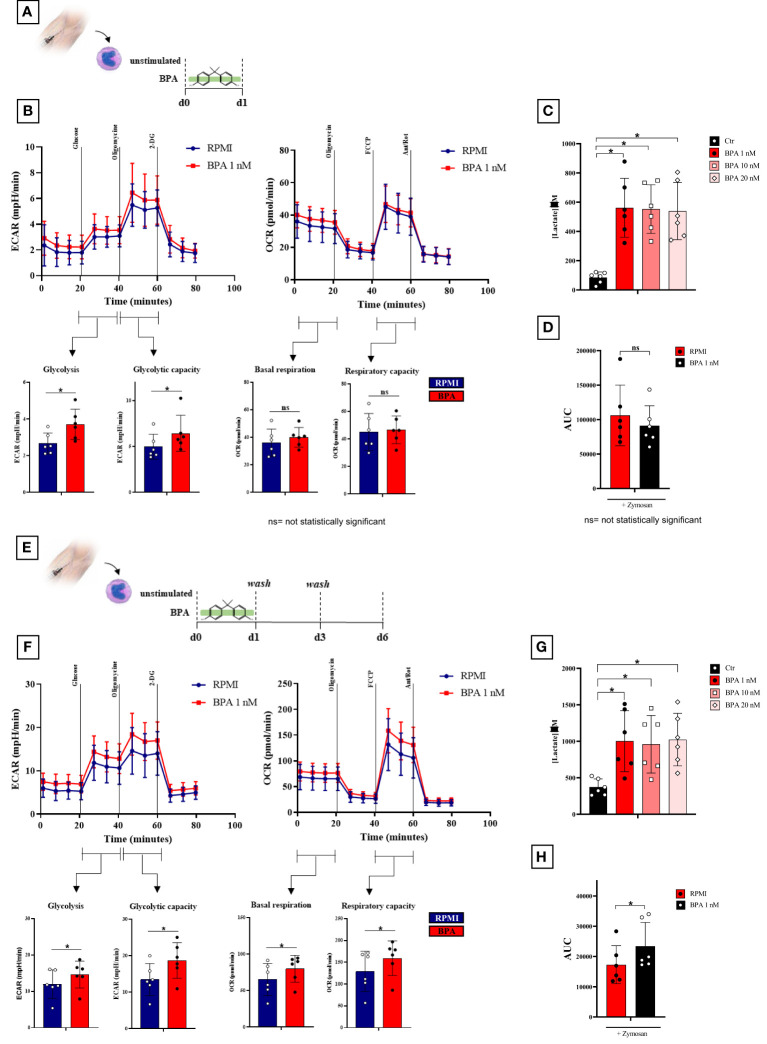
BPA induces a rewiring of the immune metabolism after acute stimulation and trained immunity induction. **(A)** Schematic of *in vitro* BPA acute experiments. **(B)**, Seahorse metabolic flux analyses of glycolytic (left) and mitochondrial (right) metabolism after BPA acute stimulation. **(C)** Extracellular lactate production after 24 h BPA stimulation. **(D)**, ROS levels in monocytes pre-treated for 24h with BPA and then stimulated with 10 mg/mL zymosan. **(E)** Schematic of *in vitro* trained immunity experiments. **(F)** Seahorse metabolic flux analyses of glycolytic (left) and mitochondrial (right) metabolism in BPA-trained cells. **(G)** Extracellular lactate production from day 3 to day 6 in BPA-trained cells. **(H)** ROS levels in macrophages trained with BPA and then stimulated with 10 mg/mL zymosan. (Wilcoxon signed ranks test *p<0.05). *BPA, Bisphenol A; ECAR, extracellular acidification rate; OCR, oxygen consumption rate; ROS, Reactive Oxygen Species; AUC, Area Under the Curve*. ns, not statistically significant.

To further confirm the BPA-induced reprogramming of monocytes we extensively investigated the metabolic features following acute stimulation and trained immunity induction (**Figure 5**). To this aim, we measured extracellular lactate after 24h and from day 3 to day 6 in the training protocol. Our data showed a significant increase in lactate secretion both after 24h monocytes stimulation (**Figure 5C**) and at day 6 in BPA-trained macrophages (**Figure 5G**) in all the concentrations tested compared to the controls, suggesting a metabolic switch towards a glycolytic metabolism. However, no significant differences in lactate secretion were highlighted between the different BPA concentrations, therefore 1 nM was chosen for further experiments. In order to delve deeper into this indication, we carried out Seahorse metabolic flux analyses to probe glycolytic and oxidative metabolism of BPA acutely stimulated cells after 24 h, BPA-trained cells at day 6 and each unstimulated controls. BPA acute stimulation induced significantly higher basal glycolysis and glycolytic capacity (**Figure 5B**, left), while no differences were highlighted in the oxidative metabolism compared to the control (**Figure 5B**, right). Accordingly, no differences in ROS production were detected in BPA-treated cells compared to the control (**Figure 5D**).

More interestingly, BPA training had a marked effect on metabolic parameters measured on day 6, with a significant higher basal glycolysis and a significant increase of oligomycin-triggered maximum glycolytic capacity (**Figure 5F**, left). Additionally, both baseline- and carbonyl cyanide-p-trifluoromethoxyphenylhydrazone-triggered maximum respiration rates were significantly augmented by BPA training (**Figure 5F**; right) along with an increase in ROS production (**Figure 5H**).

### 3.5 BPA did not affect cell viability

**Figure 6:**
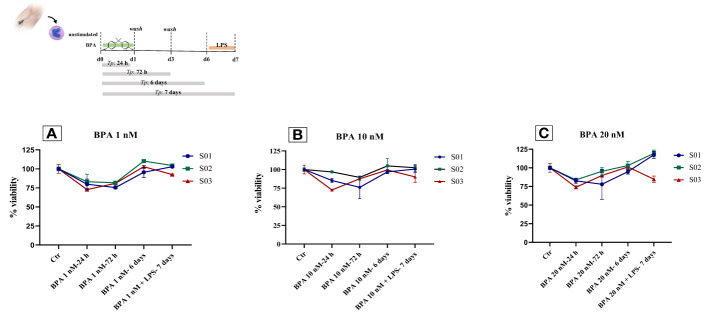
Monocytes viability after BPA 1 nM **(A)**, 10 nM **(B)** and 20 nM **(C)** stimulations: Time-course dose-dependent MTT assays. *BPA, Bisphenol A; Ctr, controls; LPS, Lipopolysaccharide*.

We assessed cell viability with an MTT assay to show the effect of BPA at each concentration used for this purpose. We performed a time-course and dose-response experiment measuring the cell viability at 4-time points (24h, 72h, 6 days, and 7 days) after the BPA stimulation. The stimulation with BPA 1 nM had no strong effects after 24 hours, resulting in a cell viability rate of 78.59% and a mortality rate of 21.41%, similar to the results obtained after 72 hours (79.3% viability, 20.7% mortality rate), compared to unstimulated cells. Interestingly, after 6 days of rest from BPA 1 nM treatment, we found an increase in the cell viability of 2.88%, compared to controls. This percentage of viability was maintained also at day 7, after the restimulation with LPS (**Figure 6A**). Despite the stimulation with a higher concentration of BPA (10 nM), the cell viability, after 24h and 72h, remained similar (84.7% viability, 15.3% mortality rate) and even higher than the viability with BPA 1nM. Furthermore, after 6 days of rest from BPA 10 nM treatment cell viability was slightly augmented by 1%, compared to control cells. We obtained the same results also after the restimulation with LPS, on day 7 (**Figure 6B**).

At the final concentration of BPA 20 nM, the cell viability rate after 24 hours and 72 hours of treatment was 80.1% and 87.7% with a mortality rate of 19.9% and 12.3%, respectively, compared to untreated cells. After 6 days of rest, the cell viability rate was equal to the controls. On day 7, after the restimulation with LPS, two subjects showed an increase in cell viability of 17.5% and 19.3%, whilst only one subject exhibited a reduction in the cell viability of 15.3%, in contrast to the previous two, compared to the controls (**Figure 6C**). Additionally, to strengthen the information acquired about the cell proliferation rate, we analyzed the cell number detaching the cells at day 6 before restimulation. Subsequently, cells were reseeded and counted also at day 7 after restimulation with LPS, and we observed an overall rise in cell number of about 5%, compared to the controls.

### 3.6 Correlation analysis

**Figure 7:**
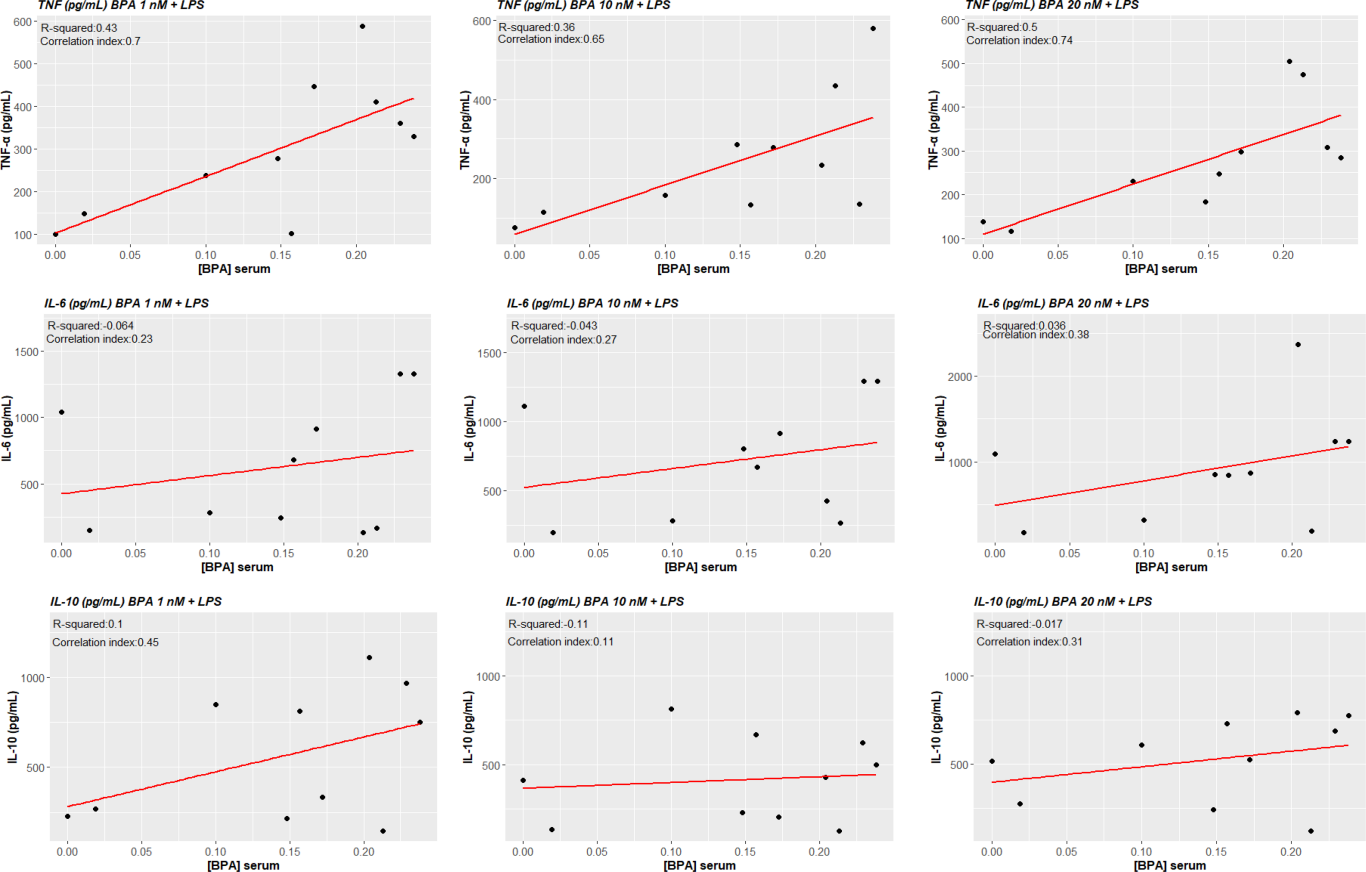
Correlations between cytokines levels BPA-trained cells’ production and circulating serum levels of BPA. Pearson correlations show a statistically significant association between ex vivo TNF-α production of BPA-trained cells and circulating serum levels of BPA (p<0.05), while no statistical significance was reached for IL-6 and IL-10. *BPA, Bisphenol A; TNF-a, Tumour necrosis factor alpha; IL-6, Interleukin-6; IL-10, Interleukin-10*.

**Figure 8:**
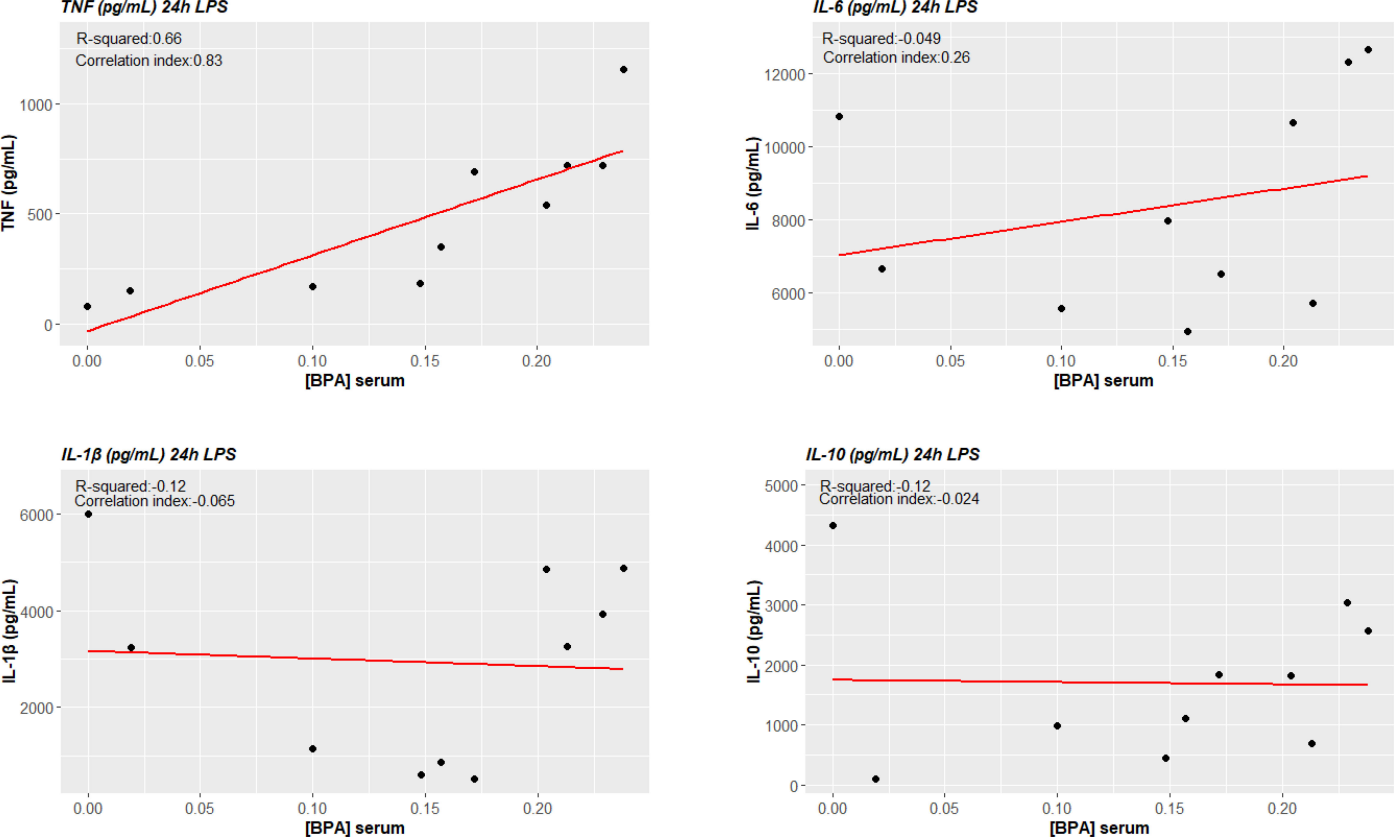
BPA-mediated trained immunity *in vivo*: TNF-α production after LPS acute stimulation correlates with circulating serum levels of BPA. Pearson correlations show a statistically significant association between ex vivo TNF-α production of LPS acute stimulated cells and circulating serum levels of BPA (p<0.05), while no statistical significance was reached for IL-6 and IL-10. *BPA, Bisphenol A; TNF-a, Tumour necrosis factor alpha; IL-6, Interleukin-6; IL-10, Interleukin-10*.

TNF-α production in ex vivo BPA-treated monocytes at each concentration was significantly associated with circulating concentrations of BPA (BPA 1 nM + LPS cor. index 0.703, p-value 0.023; BPA 10 nM + LPS cor. index 0.655, p-value 0.040; BPA 20 nM cor. index 0.744, p-value 0.014). Conversely, IL-6 and IL-10 did not significantly associate with serum concentrations of BPA (**Figures 7**). Additionally, we tested the possibility of a BPA-mediated training phenotype *in vivo* performing correlation analysis between cytokine response after LPS acute stimulation and BPA serum levels. TNF-α production again resulted significantly associated with circulating concentrations of BPA (cor. index 0.835, p-value 0.003), while IL-6, IL-1β and IL-10 did not significantly associate (**Figure 8**).

## 4 Discussion

The increased environmental pollution can lead to the accumulation of pollutants in several tissues in the human body and can induce inflammation or even cause disease (18–22). The continuous accumulation and long-term effects of these molecules in immune responses are largely unknown. Recently, several studies reported the association between environmental BPA exposure and immunologic-related diseases (18–20). BPA is a well-known EDC that can interfere with endocrine balance (23). Particularly, the exposure to low doses may be related to different biologic effects in comparison to high doses, determining a variable dose-response relationship. The most relevant BPA metabolic and immunological effects occur in the case of low-dose exposure, while they are not observed by using higher concentrations (24).

In our study, we shed light on the role of BPA as a novel TI-inducing factor in human primary monocytes. Considering the BPA accumulation in several human tissue reservoirs such as adipose tissue, we demonstrated its serum detectability in nanomolar levels from a healthy cohort of individuals. In recent research carried out by Meslin et al., the authors highlighted the risk of toxicity related to low-dose exposure in a European cohort of individuals, although the tangled network of mechanisms underlying its effect still remains largely unclear (25). Based on these findings, we tested if different concentrations (1 nM, 10 nM, and 20 nM) of BPA widely considered as low concentrations and in line with circulating serum concentrations could induce TI *in vitro* (16, 26, 27). Moreover, we assessed the BPA direct effect in terms of cytokines production after 24 hours of acute stimulation including LPS as positive control. Data showed a slightly increased in pro-inflammatory cytokines TNF-α and IL-1β, while IL-6 resulted in higher expression together with the anti-inflammatory cytokine IL-10. Nevertheless, comparing the quantity of cytokines produced by BPA and LPS stimulations, it is possible to figure out that BPA is not a strong stimulator in the acute phase. Taken together, these data further confirm the widely described role of BPA at low concentrations in the establishment of low-grade inflammation (13, 28–30). However, chronic exposure to low concentrations of BPA could lead to functional changes in different cell types.

In our setting, following the optimal protocol for the detection of the trained response, we trained the primary monocytes isolated from venous blood samples with BPA for 24 hours (training period) with a consequent resting period of 6 days, before the re-stimulation with LPS. BPA demonstrated a key role as a priming stimulus in the establishment of a trained phenotype. The induction of TI by BPA exposure could open the way for the study of the role of environmental pollutants in the long-term modulation of innate immune cells. Herein, we describe a new protocol of TI in which BPA demonstrated a key role as a priming stimulus in the establishment of a trained phenotype.

In all experimental conditions, our data show the increase of both pro-inflammatory (TNF-α and IL-6) cytokines highlighting TI features. Moreover, it is important to point out that the effects of TNF-α are more striking even for low concentration. Additionally, we found an increase in the anti-inflammatory IL-10 production possibly due to a compensative phenomenon, characterizing healthy subjects, that prevents overshooting inflammation and tissue damage by limiting the inflammatory response in time. These results further support the hypothesis of a strong immunological impact of BPA at low doses commonly found in the circulation of healthy volunteers.

Importantly, correlation analysis between TNF-α production after BPA-mediated TI and BPA serum concentrations showed that these parameters are significantly associated, linking *in vitro* and *in vivo* data. In addition, we explored the possibility if the cells were already to some extent trained *in vivo* by BPA. Interestingly, we found the same correlation between TNF-α production after LPS acute stimulation and BPA circulating levels.

Cellular metabolism is a critical mediator of the trained immunity-dependent reprogramming of innate immune cells (31–33). Indeed, the rewiring of the immunometabolic circuits is reflected in the profound changes in cellular metabolic pathways such as glycolysis and oxidative phosphorylation, increasing the capacity of the innate immune cells to respond to a secondary stimulation (33, 34).

To this aim, we broadly investigated the possibility of BPA-induced changes in the immune metabolism. Particularly, we observed a metabolic switch towards an aerobic glycolytic metabolism after acute stimulation, which can be explained with the acute need of rapid energy production in the low-grade inflammatory context BPA-mediated (35). More noteworthy, here we showed that BPA-induced trained immunity enhances not only glycolysis with a higher lactate production, but also oxygen consumption and increased ROS levels pointing that trained cells use different metabolic pathways to adapt their function to produce energy faster and more efficiently.

Several lines of evidence in the trained immunity field highlighted the key role of the aerobic glycolytic metabolism as a hallmark of β-glucan and BCG-trained cells (36, 37). However, several studies investigated the role of TCA cycle and OXPHOS in trained immunity revealing that the TCA cycle remains function for ATP production by OXPHOS as demonstrated by the augmented basal and maximum oxygen consumption rate on day 6 before restimulation of the cells (38, 39).

Additionally, to give further relevance to the effects induced by BPA, we proved that these concentrations of BPA do not have toxic effects on cell viability. Altogether, the treatment with each concentration employed showed only a slight reduction in cell viability (~ 20%) after 24 hours, as already shown in a macrophage mouse cell line by Huang et al., 2018 (40). In addition, when BPA stimulus was removed by one washing step, cell viability was also ~ 80% 72 hours later. Interestingly, the resting period and the re-stimulation influence positively the cell growth, determining an increase in the cell proliferation rate, as demonstrated also by Camarca et al. (26) These results provide us a reason to state that BPA does not affect cell viability and further support the functional changes induced by BPA observed in this study.

Recently, Wang et al. (41) demonstrated the role of BPA in inducing RNA and protein overexpression of TLR-4/NF-κB pathway and downregulating IκBα both *in vivo* and *in vitro* experiments. Interestingly, the same molecular mechanisms (TLR/MyD88 activation) have been found at the basis of trained immunity induction in macrophages in an *in vivo* experiment on mice, in which knock-out mice MyD88-/- did not show trained immunity phenotype (42).

In conclusion, our study demonstrates that the environmental chemical compound BPA has important immunological effects. In particular, we describe the role of BPA as a stimulus that can induce Trained Immunity, demonstrating that even low doses of this endocrine disrupter can affect immune cells of healthy individuals. Inappropriately activated trained immunity responses can contribute to pathogenesis of inflammatory diseases, resulting in either a chronic hyper-inflammatory state or a persistent state of immunological tolerance. Considering this double role of trained immunity, it is reasonable to consider BPA as a potential driver of trained immunity dysregulation, especially in combination with other insults over lifetime, leading to different pathologies.

## Data availability statement

The original contributions presented in the study are included in the article/**Supplementary Material**. Further inquiries can be directed to the corresponding author.

## Ethics statement

The studies involving humans were approved by The University of Campania “L. Vanvitelli” in Naples (protocol N17234/2022). The studies were conducted in accordance with the local legislation and institutional requirements. The participants provided their written informed consent to participate in this study.

## Author contributions

MD: Conceptualization, Formal Analysis, Investigation, Methodology, Writing – original draft. LV: Conceptualization, Formal Analysis, Investigation, Methodology, Writing – original draft, Writing – review & editing. MR: Conceptualization, Formal Analysis, Investigation, Methodology, Writing – original draft, Writing – review & editing. FS: Data curation, Investigation, Resources, Visualization, Writing – review & editing. ND: Conceptualization, Data curation, Project administration, Supervision, Writing – review & editing. MM: Data curation, Investigation, Resources, Visualization, Writing – review & editing. MC: Data curation, Investigation, Resources, Visualization, Writing – review & editing. AC: Data curation, Investigation, Resources, Visualization, Writing – review & editing. AZ: Data curation, Investigation, Resources, Visualization, Writing – review & editing. MN: Conceptualization, Data curation, Project administration, Supervision, Writing – review & editing. AF: Conceptualization, Data curation, Project administration, Supervision, Writing – review & editing.
