# Supplementary material for: Environmental bisphenol A exposure triggers trained immunity-related pathways in monocytes
Source: Front Immunol. 2023 Nov 23;14:1270391. doi: 10.3389/fimmu.2023.1270391 (PMC10701735; doi:10.3389/fimmu.2023.1270391)
Supplement: Supplementary file 1 [file DataSheet_1.pdf]

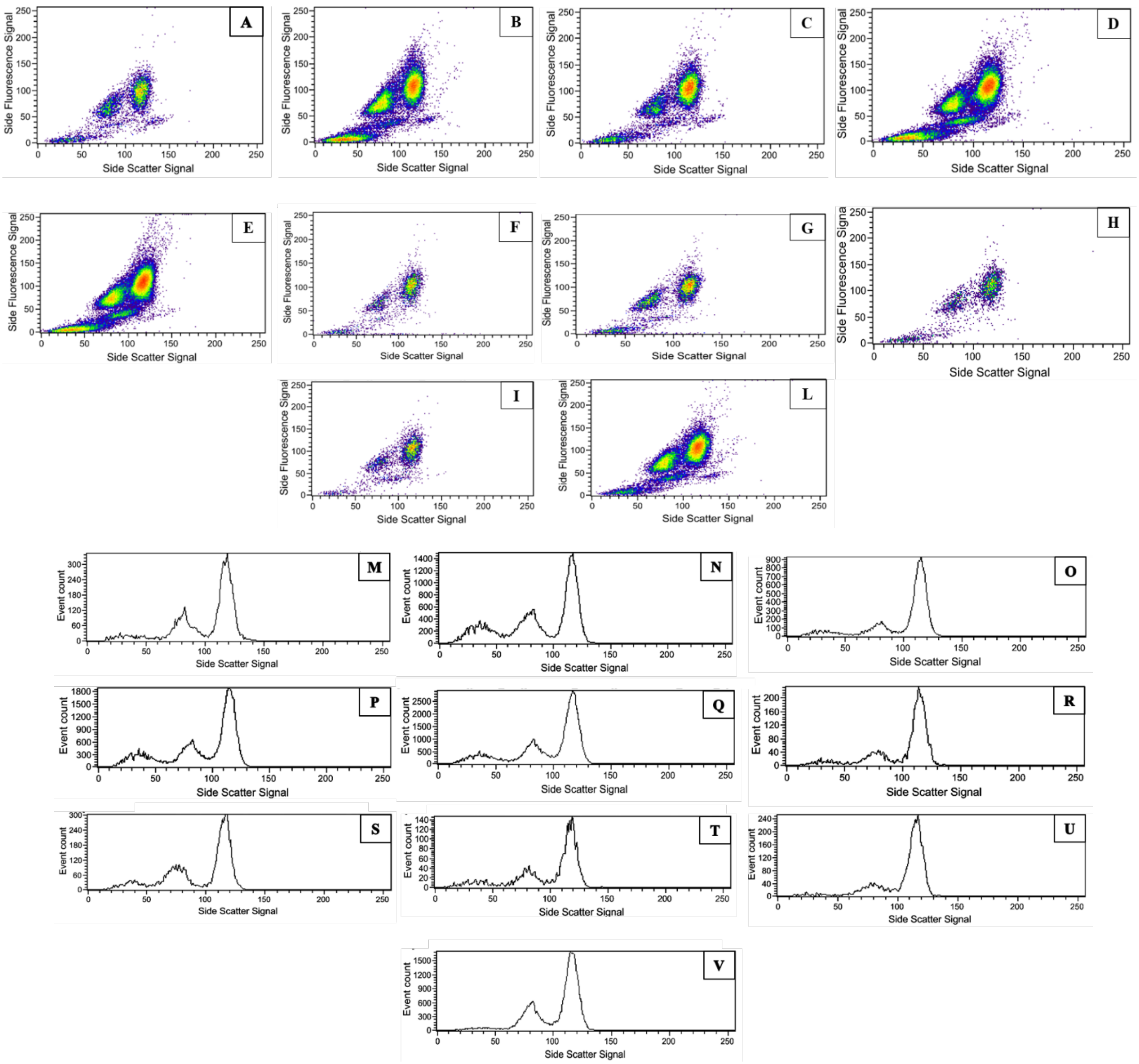

***Fig. 1** Monocytes purity after Percoll isolation. Density plots (A-L) and Histograms (M-V) confirmed the enriched monocytes-suspensions isolated from the 10 healthy donors.*

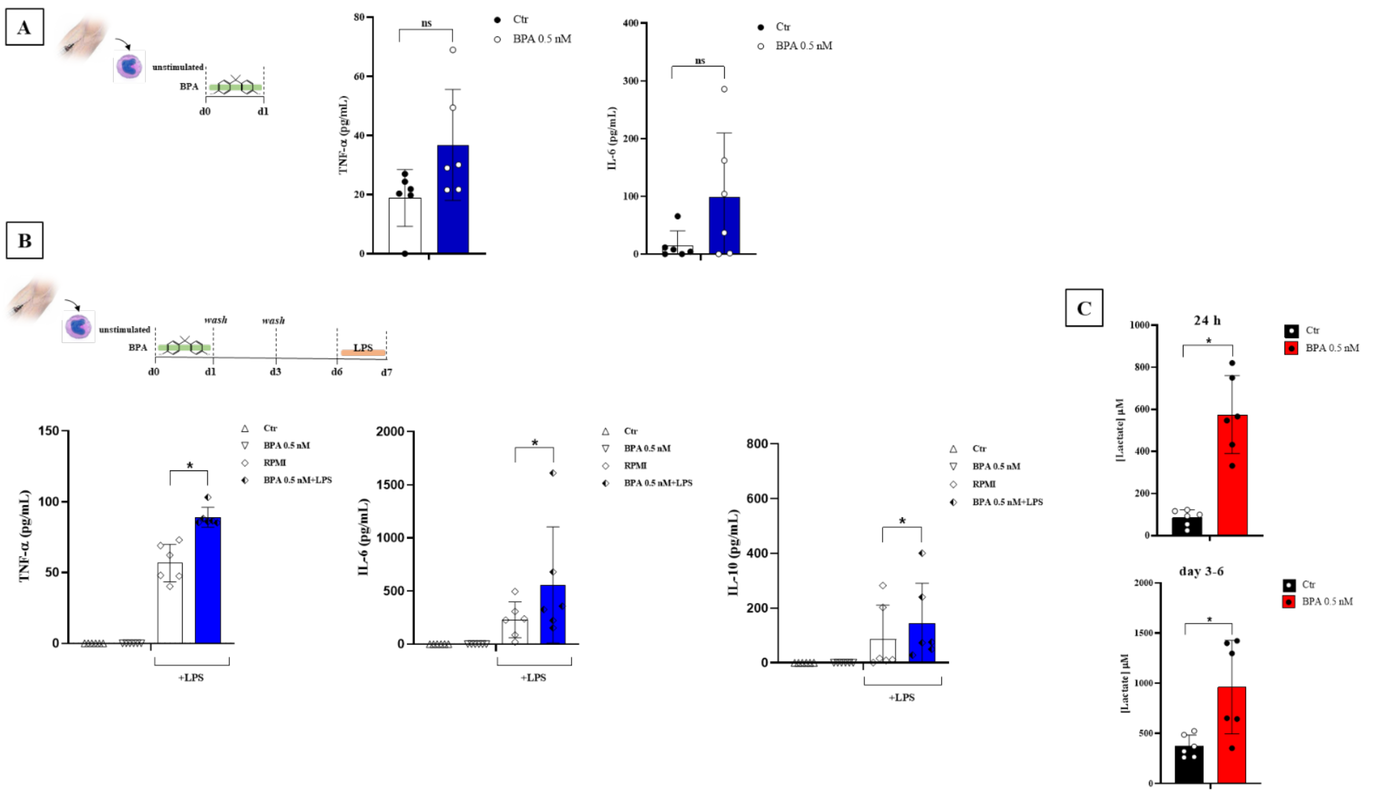

Fig. 2 *BPA lowest dose still induces cytokines production after acute stimulation and trained immunity induction. Pro-inflammatory and anti-inflammatory cytokines [Tumour necrosis factor alpha; Interleukin-6 and Interleukin-10] production after BPA 0.5 nM acute stimulation (A) and training (B). Extracellular lactate production after BPA 0.5 nM 24h stimulation and day 3 to day 6 of training protocol (C). (Wilcoxon signed ranks test *p<0.05; **p<0.005; ns: not significant). BPA: Bisphenol A; TNF-a: Tumour necrosis factor alpha; IL-6: Interleukin-6; IL-10: Interleukin-10; Ctr: controls; LPS: Lipopolysaccharide*
